# Genomic changes of Lassa virus associated with mammalian host adaptation

**DOI:** 10.1186/s12864-025-11666-y

**Published:** 2025-05-15

**Authors:** Linda Easterbrook, Xiaofeng Dong, Jack Smith, Susan Fotheringham, Sarah Kempster, Catherine Hartley, Tessa Prince, Victoria Graham, Emma Kennedy, Stephen Findlay-Wilson, Lucy Crossley, Roger Hewson, Neil Almond, Julian A. Hiscox, Stuart Dowall

**Affiliations:** 1 UK Health Security Agency (UKHSA), Porton Down, Salisbury, SP4 0JG Wiltshire UK; 2 Institute of Infection, Veterinary and Ecological Sciences, University of Liverpool, Liverpool, L3 5RF UK; 3 Medicines and Healthcare products Regulatory Agency (MHRA), Blanche Lane, South Mimms, Potters Bar, Hertfordshire, EN6 3QG UK

**Keywords:** Lassa virus, Adaptation, Passage, Mutation, Changes

## Abstract

**Background:**

Lassa virus (LASV) causes a severe haemorrhagic fever in humans, with estimates of 100,000 to 300,000 infections annually in endemic regions and accounting for around 5000 deaths. The natural reservoir is the *Mastomys* rat, but through zoonotic transmissions humans are accidental hosts. Regular outbreaks continue to exert pressures on public health systems, with its ability to cause nosocomial infections posing risks to healthcare workers. It is a concern that larger outbreaks and introduction of LASV to new territories will intensify, including risk of adaptation to new mammalian host reservoirs.

**Results:**

To evaluate genetic changes in LASV during adaptation to a new host, a guinea pig model of infection was utilised. Initial infection with LASV stocks cultured from cell culture resulted in only mild or subclinical disease. To study the susceptibility in naïve animals, the virus was serially passaged which increased clinical signs during disease progression ultimately resulting in severe disease. An RNAseq and consensus mapping approach was undertaken to evaluate nucleotide changes in LASV genome from each animal at each passage.

**Conclusions:**

During adaptation to guinea pigs, no significant new mutations occurred. Instead, a selection pressure on two genes of the L segment was observed resulting in their increased frequency in the genome population during passaging.

## Introduction

Lassa Haemorrhagic Fever (LHF) is a severe infection caused by *Mammarenavirus lassaense* (LASV), a member of the *Arenaviridae* family. The virus is often cited as being responsible for between 100,000 and 300,000 infections annually in endemic regions, with around 5000 deaths [1, 2]. However, a group including 20 international LASV subject matter experts state that the true numbers remain unknown and may even be underestimated [3]. The majority, approximately 80%, of Lassa fever patients experience asymptomatic or mild illness, recovering within 10 days [4]. Severe haemorrhagic fever, requiring hospitalisation, manifests with encephalitis, bleeding (especially from the gums, nose and eyes), respiratory distress, vomiting, shock and organ failure in 20% of cases [5]. In hospitalised cases, mortality is between 15 and 20% [1]. As with all mammalian Arenaviruses, LASV is a rodent-borne virus. The *Mastomys* rat is the natural reservoir of LASV and humans are an accidental host. Zoonotic transmission occurs through direct contact with rodent excreta, inhalation of contaminated dust and ingestion of contaminated rodent meat [6]. Person-to-person transmission occurs in a similar pattern to other viral haemorrhagic fevers (VHFs) such as Ebola [7], via shedding in human body fluids from an infected patient [8]. In addition, the virus is present in semen [9], posing significant risk of sexual transmission, as has been documented for *Orthoebolavirus zairense* [10]. A mapping study has suggested as many as 37.7 million people, across 14 countries in West Africa are at direct risk of exposure to Lassa virus [11]. There is no approved vaccine or treatment for LASV and it is included on the World Health Organisation (WHO) list of priority pathogens [12]. The high risk of transmission, accompanied by severity of disease and lethality has resulted in LASV being classified as a hazard group 4 (HG4) pathogen– alongside other members of the *Mammarenavirus* genus.

The *Mammarenavirus* genus is monophyletically classified into two geographical groups, Old World and New World. The Old World viruses are geographically located across Africa and include LASV, Lujo virus (LUJV) and Lymphocytic Choriomeningitis virus (LCMV). The larger New World complex is further split into three clades (A, B and C). Clade B is the most clinically important, containing several HG4 VHF causing viruses, such as Junin virus (JUNV), Machupo virus (MACV) and Guanarito virus (GTOV). There are four confirmed genetic lineages of LASV, clustered regionally across West Africa (Lineages I-IV) and an additional three lineages which have been discovered but are yet to be fully classified (Lineages V-VII) [6]. Of the seven lineages, human infection is most commonly associated with Lineage II and IV [13].

The LASV genome is a single stranded, ambisense, bi-segmented RNA. The large (L) and small (S) segment combined, encode for four viral proteins in ambisense manner [6]. The L segment encodes the L protein and the Z protein, whilst the S segment encodes the viral glycoprotein (GP) precursor and the nucleoprotein (NP). The L protein, an RNA-Dependent RNA Polymerase (RDRP), works with the NP to mediate genome transcription and viral replication. The Z protein (a zinc-finger motif) engages in multiple essential functions throughout the viral life cycle including orchestration of viral assembly, budding and antagonisation of the host type I-interferon [14, 15]. Viral replication and host immunity modulation is supported by the NP. The NP encompasses the viral genome as the primary structural component of LASV. As with all Mammarenaviruses, entry to the host cell is mediated by the GP.

Animal models are a crucial tool for effectively understanding the mechanisms of infectious diseases, as well as being essential to the development of medical countermeasures such as vaccines. Previous studies have demonstrated that Hartley guinea pigs are not uniformly susceptible to LASV [16, 17], often– but not always [18, 19] - requiring viral adaptation through forced evolution to develop a successful model. This enables close observation of clinical changes observed within guinea pigs as the virus becomes more adapted to the host, as well as a chance to understand genetic drift as LASV adapts to a new host. Additionally, RNAseq analysis of the virus as it adapts enables assessment of the ability of the virus to alter key structural proteins to facilitate infection of a host.

## Results and discussion

### Adaptation of Lassa virus to guinea pigs

LASV, like Ebola virus [20], is often initially non-pathogenic in guinea pigs. A forced evolution model enables investigation on genomic changes with increasing pathogenicity as the virus is sequentially passaged in vivo using a guinea pig model. The virus becomes increasingly virulent and adapted to replicating within the host, with increased lethality.

**Fig. 1:**
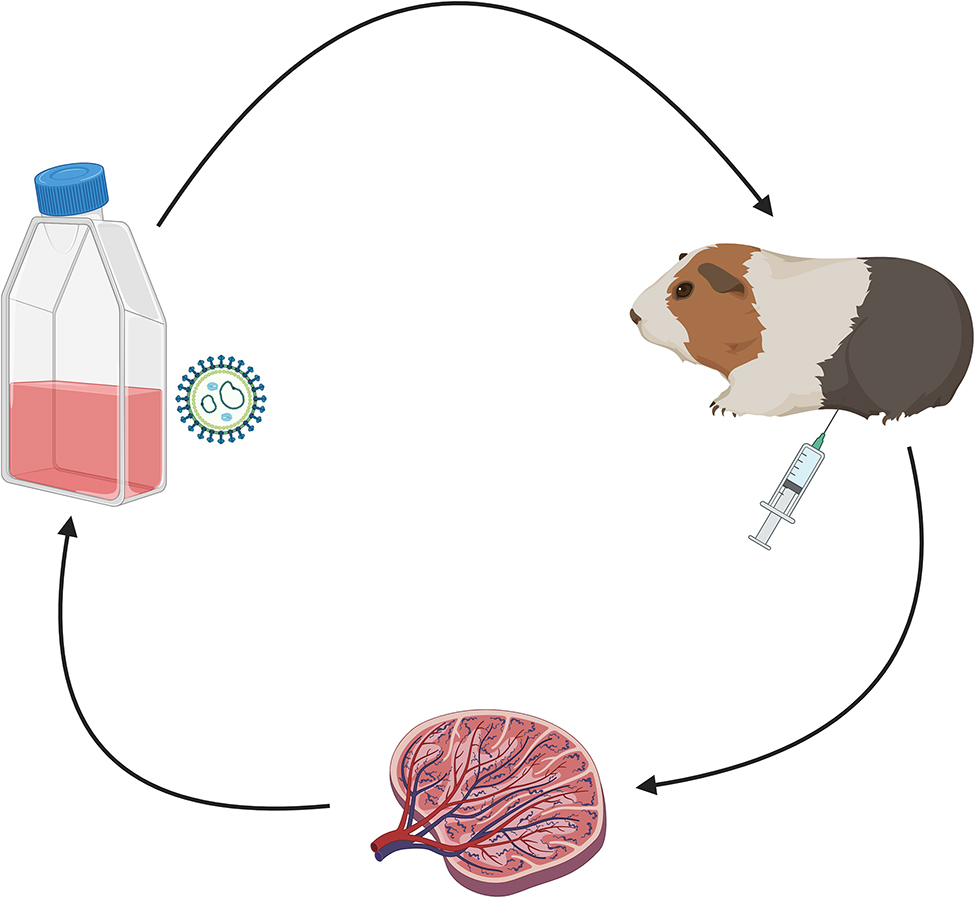
Passaging of Lassa virus in vivo. Lassa virus was passaged through guinea pigs five times in order to increase virulence and increase lethality in a forced evolution model. There were 10 animals per group of which five were used at each passage to harvest LASV for the subsequent infection. The remaining five animals were observed for clinical manifestations of disease and to assess lethality of the virus

Guinea pigs were infected with 10^4^ TCID_50_ LASV and the virus was then isolated and serially passaged to develop uniform lethality in the guinea pig model (Fig. 1). Each passage contained 10 guinea pigs, five of which were culled at day 7 (passages 1–2) or 14 (passages 3–5) to obtain spleens and virus for the subsequent passage. Earlier timepoints were chosen in the first passages to reduce the risk that virus would be cleared by the day 14 timepoint, thus ensuring sufficient amounts of live virus for the subsequent infection studies. Five guinea pigs were used to observe clinical parameters at each passage for up 21 days post-challenge. Sequential infection of guinea pigs was continued until evidence of clinical and virological adaptation of LASV to guinea pigs was observed.

### Clinical observations

**Fig. 2:**
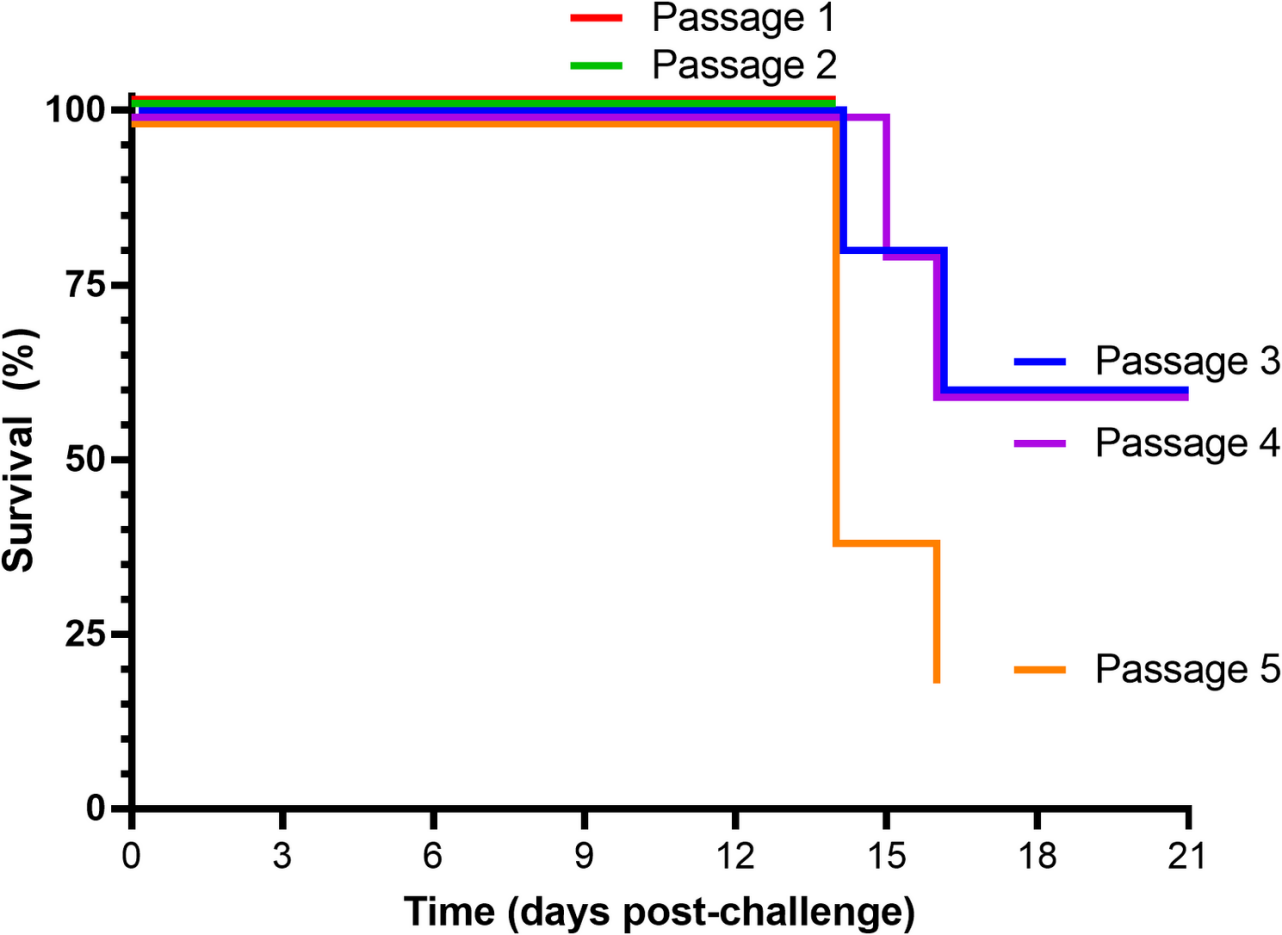
Survival of guinea pigs after challenge with passaging of LASV. Kaplan-Meier survival plot showing animals that had reached humane clinical endpoint criteria with adaption of the virus to the guinea pig host

**Fig. 3:**
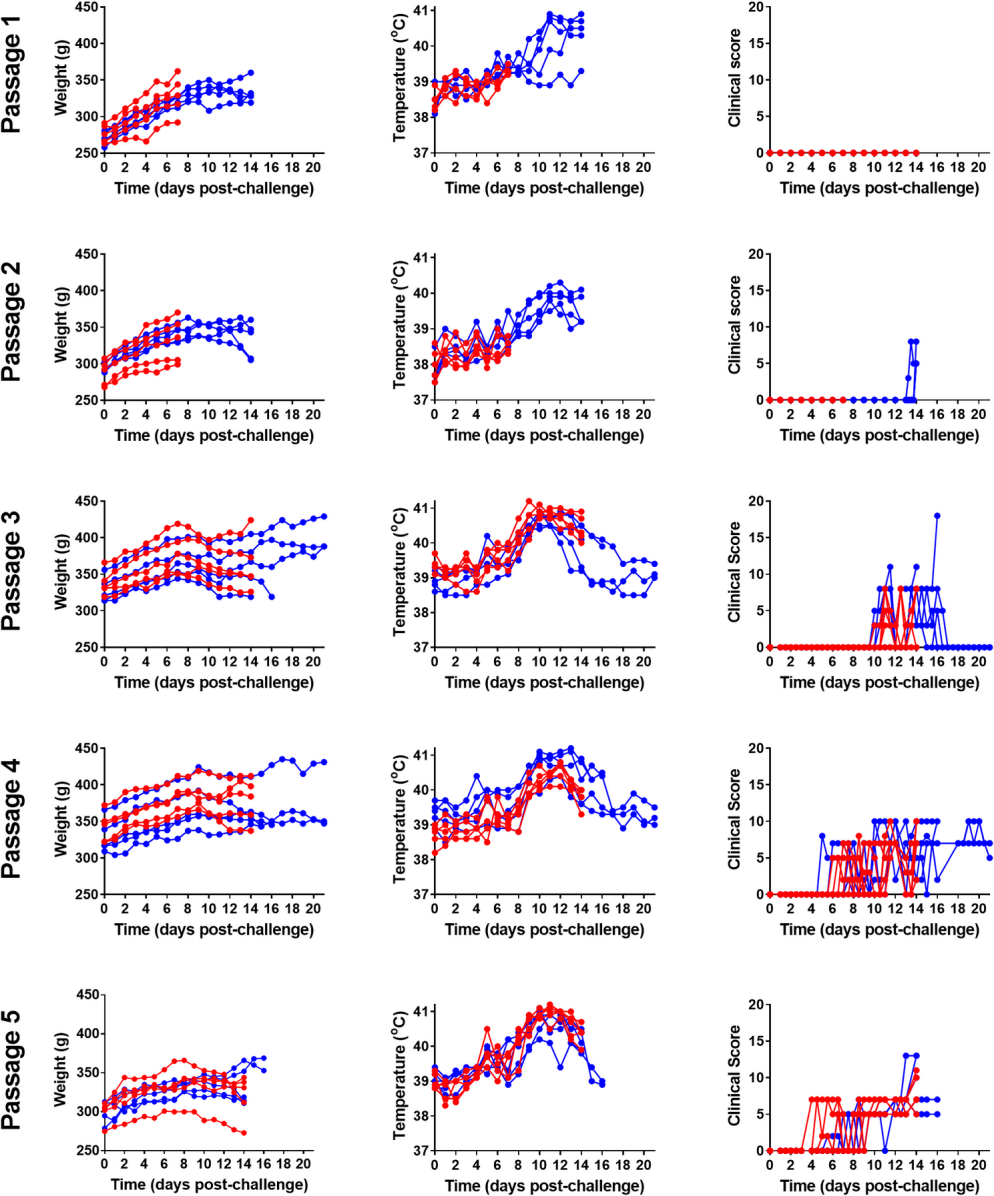
Clinical details of guinea pigs post challenge with sequential passages of Lassa virus (LASV). Weight, temperature and clinical observations were recorded over the course of each study to determine the pathogenicity and virulence of subsequent passages of LASV. 10 guinea pigs were challenged in each study. Five guinea pigs were culled early in order to isolate passaged virus (indicated in red) and five continued until day 14 or 21, to assess clinical signs of disease and observe virological changes

Guinea pigs were challenged with LASV GA391 strain and the virus was serially passaged with the aim of developing uniform lethality in the guinea pig model. Subsequent passaging of LASV through guinea pigs led to adaptation of the virus to the host, as evidenced by survival and clinical outputs (Figs. 2 and 3, respectively). Clinical observations and survival are the key initial parameters to determine the progression of disease in LASV-challenge guinea pigs, so reported analysis from the animal studies has been restricted to these outputs. Challenge with passage one and two resulted in limited to no clinical illness and 0% lethality. Passage three challenge was the first to induce clinical disease in the guinea pigs, with signs included ruffled fur, arched, lethargy, wasp-waisted, unsteady gait and rapid breathing alongside a 40% lethality rate. Passage three also marked the first disease course with the characteristic increase of body temperature exceeding 40ºC, rapidly declining before death, as seen in other LASV guinea pig models [21]. Subsequent challenges with passage four and five resulted in increased clinical manifestation, with recorded signs including those seen at passage 3 in addition to behavioural changes. The lethality remained at 40% between passage three and four, despite more animals exhibiting clinical signs of infection. Challenge with passage five stock resulted in similar clinical manifestation alongside an 80% mortality rate. It is worth noting that the passage five study was culminated early for the purpose of animal welfare on recommendation from the named animal care and welfare officer (NACWO) once 4/5 guinea pigs had reached humane endpoint; however, all animals experienced characteristic temperature change and clinical signs of LASV infection and as such it is likely the final animal would have reached humane endpoint shortly after. In those animals that met humane clinical endpoints, this occurred at 14–16 days post-challenge.

Our results are in contrast to those published by Clegg and Lloyd, who used the same GA391 LASV strain, challenge dose and breed of guinea pigs with similar weights and showed uniform lethality of unvaccinated animals without adaptation [18]. Unfortunately, details on how the virus was grown and characterised are not provided, thus preventing a direct comparison. Whilst specific details on the challenge stock are scant in the publication from 1987, it is was likely to be a lower passage than that used in our study, with passage 5 being the lowest passage material available. Propagation of other viruses, e.g. Puumala, in Vero cells have been shown to alter susceptibility for infections of the natural reservoir, bank voles [22]; therefore, culturing conditions contribute to in vivo susceptibility.

### Genetic changes to Lassa virus

To understand the changes to pathogenicity and virulence of LASV, genetic sequencing was undertaken from each individual animal culled for spleen collection from the sequential passaging studies to ascertain occurrence of variations in the consensus genomes and determine whether they become established. Seven nucleotide changes occurred across the bi-segmented consensus genome of LASV; five loci on the L-segment and two on the S-segment (Table 1). Of these, three resulted in amino acid changes (Table 2). When another clade III LASV strain, NML-61, was adapted to guinea pigs, two mutations with higher than 50% frequencies were detected; one in the L-segment (position 1221) and one in the S-segment (position 228) [21].

Two of the amino acid changes were observed within the coding region for the L protein, which encodes the viral RNA-dependent RNA polymerase (RDRP) and a Z protein with functions of a matrix protein including orchestrating virus budding [23]. The amino acid change at position 1550 sits adjacent to the central RDRP region [24, 25] in the PB1-like domain [26]. The change at position 1939 is within the PB2-like region [24], in an area identified as a putative cap-binding domain [26] which has been reported to be involved with mediating the mRNA synthesis process [27]. The third amino acid change was in the S protein which encodes the virus nucleoprotein and precursor glycoprotein involved with virus receptor recognition and cell entry via endocytosis [23]. The position of the change at 411 is within the GP2 glycoprotein region [28], a class one fusion protein with the change specific in the membrane proximal external region [29].

**Fig. 4:**
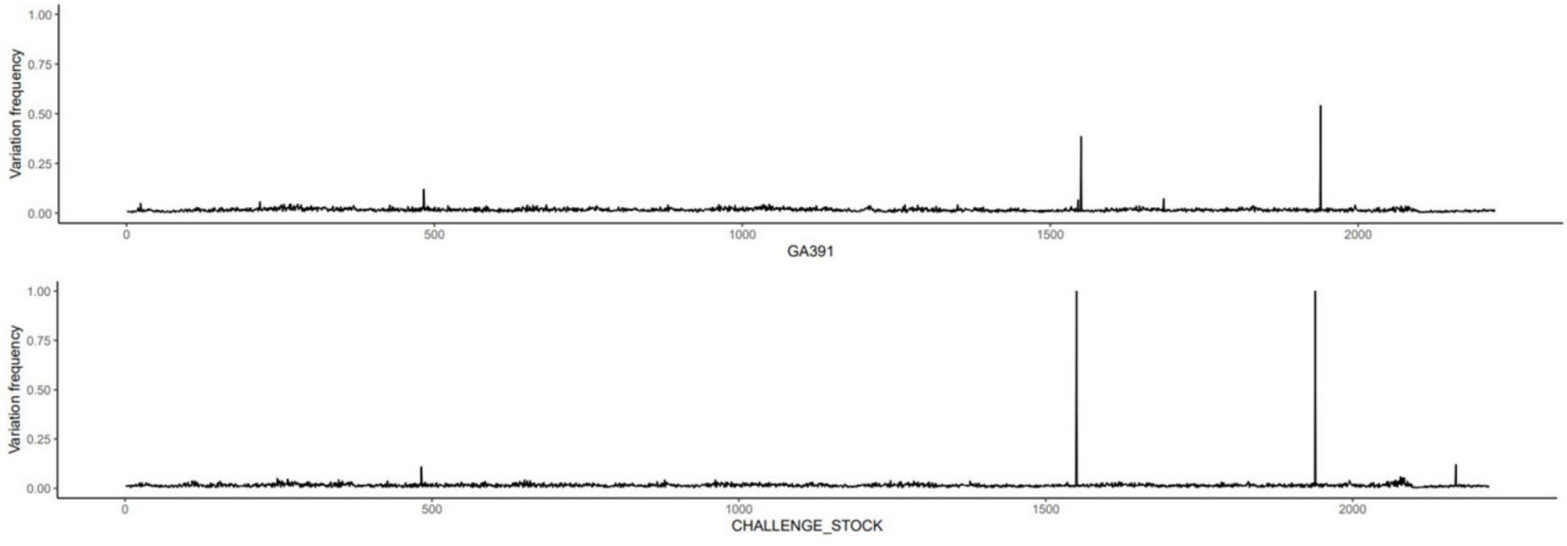
Variation frequencies in the L genome of LASV. LASV GA391 stock for initial inoculum (top); compared with guinea pig adapted stock after five passages (bottom)

The variation frequencies of amino acid on the L-segment of the genome with the initial GA391 stock were compared with the guinea pig-adapted challenge stock (Fig. 4). It was evident that the major changes of amino acid found in the adapted challenge stock had already occurred as minor changes in the initial GA391 virus preparation, with a selection pressure for two genes with increased frequency in the population. Both genes were located on the L-protein encoding segment of the genome.

**Fig. 5:**
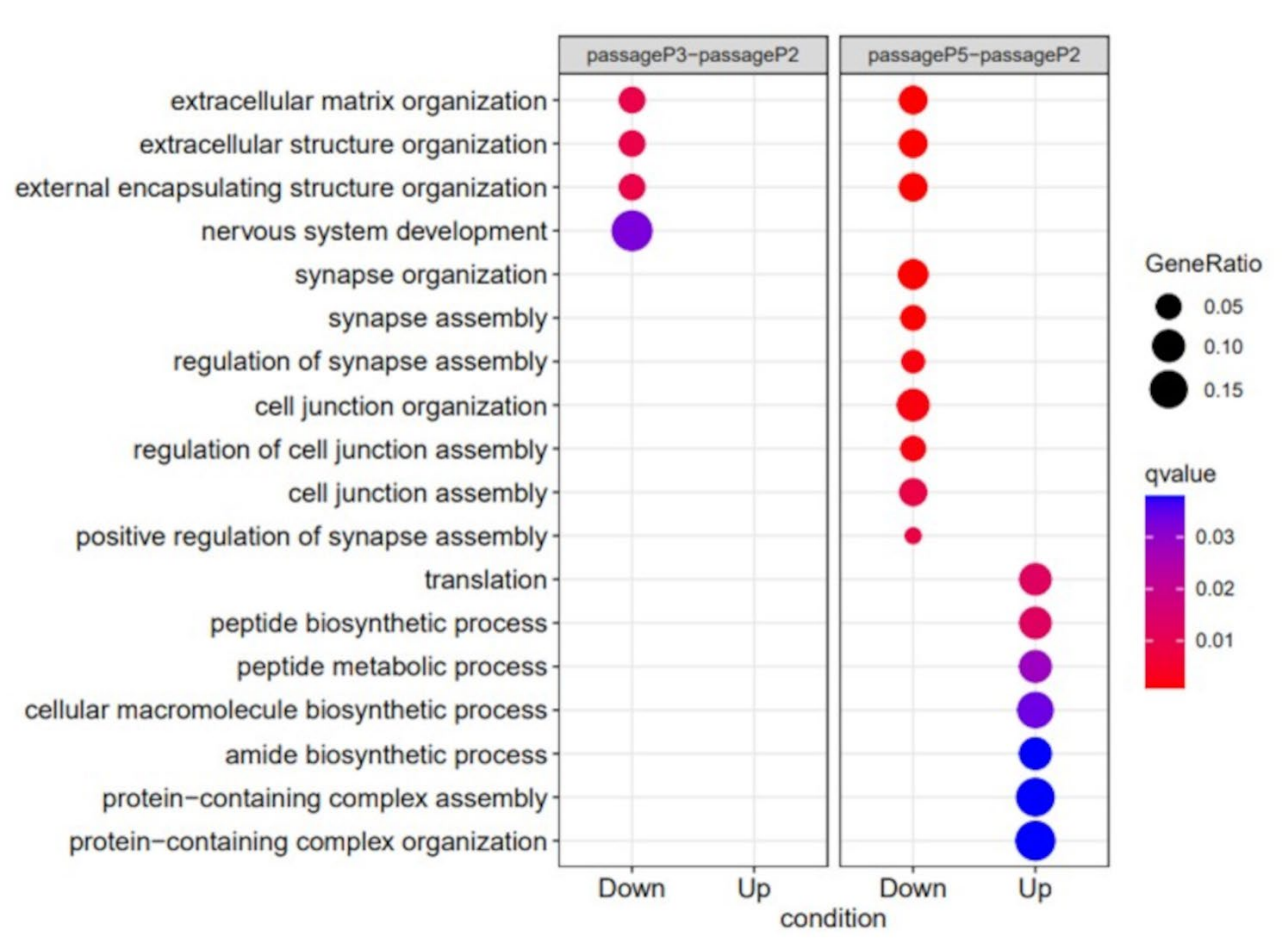
Differential expressed genes in LASV challenged guinea pigs using during passages two and five of the adaptation process

When considering the impact of adapting LASV on the guinea pig host, the up and down regulation of genes expressed by LASV challenged guinea pigs were compared between those infected at passage two and passage five (Fig. 5). Due to all material from passage one being used for subsequent inoculation, the lowest passage number with sufficient material available for sequencing analysis was passage two. Downregulation primarily occurred in the nervous system. Evidence of sensorineural hearing loss has been described in patients with LHF [30, 31] and animal models [32], particularly in the convalescent stage. The reduction in neurological gene expression may shed light on LASV interactions within the central nervous system. Neurological symptoms are associated with Old World and New World arenavirus infections [33]; however, symptoms including coma, encephalitis and convulsions are more commonly experienced in infections with JUNV [34] and MACV [35]. In addition, the upregulation of genes associated with protein synthesis and assembly is indicative of improved viral replication within the host, leading to the increase in clinical disease and mortality associated with subsequent passages.

**Table 1:** Nucleotide changes observed during passage of LASV in guinea pigs

Sample ID		Genome and Nt position						
		L					S	
		273	773	1339	2506	4891	2072	2683
Inoculation virus (GA391)		C	A	T	A	A	G	A
Passage 2	Animal 1	C	A	C	G	A	G	A
	Animal 2	C	A	C	G	A	G	A
	Animal 3	C	A	C	G	A	G	A
	Animal 4	C	A	C	G	A	G	A
	Animal 5	C	A	C	G	A	G	A
Passage 3	Animal 1	C	A	C	G	A	G	A
	Animal 2	C	A	C	G	A	A	G
	Animal 3	C	A	C	G	A	A	G
	Animal 4	C	A	C	G	A	G	G
	Animal 5	C	A	C	G	A	G	A
Passage 4	Animal 1	C	A	C	G	A	A	G
	Animal 2	C	N	N	N	N	A	G
	Animal 3	T	N	C	N	A	A	G
	Animal 4	T	T	A	G	G	A	G
	Animal 5	T	T	C	G	G	A	G
Passage 5	Animal 1	C	A	C	G	A	A	G
	Animal 2	C	A	C	G	A	A	G
	Animal 3	C	A	C	G	A	A	G
	Animal 4	T	T	C	G	G	A	G
	Animal 5	T	T	C	G	G	A	G
Adapted challenge stock		T	T	C	G	G	A	G

**Table 2:** Resultant codon and amino acid changes observed in guinea pig adapted LASV compared to initial stock virus

Genome	Nt position	AA position	Ref Nt	Alt Nt	Ref codon	Alt codon	Ref AA	Alt AA
L	273	-	C	T	Non-coding			
	773	2127	A	T	ACT	ACA	T	T
	1339	1939	T	C	AAT	GAT	N	D
	2506	1550	A	G	TTC	CTC	F	L
	4891	207	A	G	TTG	CTG	L	L
S	2072	411	G	A	ACA	ATA	T	I
	2683	207	A	G	GGT	GGC	G	G

## Conclusions

LASV poses a significant global health risk, with large scale outbreaks increasing the potential of the virus becoming more adapted to new hosts. The herein reported development of an animal model for LASV, combined with RNAseq during adaptation, has demonstrated the ability of the virus to increase its pathogenicity and virulence through just three amino acid changes. This provides crucial information on the potential of LASV to further adapt to humans and new mammalian hosts in the event of a large scale outbreak with rapid transmission, as was seen with SARS-CoV-2 [36].

The animal model herein described is unique due to be specific to the GA391 strain of LASV. The more commonly used strains use the prototypic Josiah strain. In Hartley guinea pigs, the Josiah strain requires host-adaptation [16], as observed with GA391; however, is uniformly lethal in Strain 13 guinea pigs [37]– a species not widely available. The LF2384 strain of LASV has also been used in guinea pig model development, causing uniform lethality in Hartley guinea pigs without requiring adaptations [19]. Unlike both the Josiah and LF2384 strain, which belong to Lineage IV [19], the GA391 strain is a Lineage III genotype [38]. LASV lineage diversity is a well-known problem, especially with vaccine development [39], with the ideal vaccine candidates conferring protection against divergent lineages due to the unpredictable nature of future outbreaks and descendant of any causative strain. Therefore, establishment of this model using a lineage III strain of LASV will complement existing models with lineage IV, expanding the capability for cross-protection studies to be completed with a heterologous challenge virus to the vaccine immunogen.

## Methods

### Virus

The GA391 strain of LASV was initially isolated at Porton Down, United Kingdom. The initial challenge stock of the virus was at viral passage 5 and grown and titrated on VeroE6 cells (European Collection of Cell Cultures, UK), with the titre being 1 × 10^8^ TCID_50_/mL. Prior to each challenge, the virus was prepared and diluted to a challenge concentration of 10^4^ TCID_50_/mL.

### Animals

All procedures were performed in accordance with the United Kingdom Animals (Scientific Procedures) Act 1986 under UK Home Office project license (ref P82D9CB4B) approved by the UK Health Security Agency Animal Welfare and Ethical Review Board (AWERB). Dunkin-Hartley guinea pigs were used for animal challenge studies (Harlan Laboratories, UK). Animals were anesthetised with 1.5-2% isoflurane prior to manipulation, in an induction change until sedation was achieved. LASV infected animals were housed within an isolator under climate-controlled conditions in an animal containment level 4 (CL4) facility. 10 animals were used at each passage, which from a practical point represents the maximum number of animals able to be processed at a time. Of these, 5 were culled at day 7 (passages 1–2) or day 14 (passages 3–5) post-infection with LASV for preparation of virus and 5 were carried on and used to measure clinical parameters for each passage.

### Animal challenge

For all passage experiments, the dose delivered was 10^4^ TCID_50_ diluted in sterile PBS. A surplus concentration was made to confirm concentration via back titration in cell culture. Sedated guinea pigs were subcutaneously inoculated with virus suspension in the lower right quadrant of the back. Animals were monitored for adverse effects caused by injection after being returned to their cages until anaesthetic had worn off.

### Clinical observations

Animals were monitored at least twice daily and observations (injection site reactions, movement, breathing, food-intake, water intake and appearance) recorded for the duration of the study. Humane clinical endpoints were defined (20% weight loss, or 10% weight loss with clinical symptoms) prior to challenge, defining the parameters at which animals would be euthanised to prevent unnecessary suffering. Weights of the animals were taken daily and temperatures recorded using a pre-inserted temperature chip. Clinical observations were recorded and associated with scores to monitor severity.

### Necropsy and tissue collection

Animals were humanely euthanised by intraperitoneal injection of 200 mg/kg pentobarbital sodium. Necropsies were performed within a flexi-film isolator in the animal CL4 facility. Spleens were removed from five of ten guinea pigs at each passage and stored at -80 °C. Downstream processing was carried out in the in vitro CL4 facility. Spleens were homogenised by vigorously passing through a 500 μm cell strainer (Corning, UK) with sterile phosphate buffered saline (PBS) solution (Gibco, UK). The resultant suspension was clarified by centrifugation at 400 g for 10 min to remove cell debris. The supernatant was collected, aliquoted and stored at -80 °C. A vial was used to titre the virus by TCID_50_.

### RNA Preparation

Spleen homogenate from each of the passages was added to AVL buffer (Qiagen, UK) and ethanol/isopropanol and removed from the CL4 facility into a CL3 facility for tube transfers. RNA was isolated from the sample using the Qiagen viral RNA extraction kit as per manufacturer instructions. Confirmation of LASV-specific RNA extraction was obtained by RT-PCR. RNA was pooled from individual guinea pigs at each passage was prepared for sequencing analysis.

### Sequencing

Extracted RNA was DNase treated with Turbo DNase (Ambion) using a rigorous protocol. RNA-Seq libraries were prepared from the resultant RNA using Epi-centre ScriptSeq v2 RNA-seq Preparation kit. 50ng of RNA was used as an input and libraries were purified after 12 cycles of amplification using the AMPure XP beads. Qubit was used to quantify the libraries and size distributions assessed using the Agilent 2100 Bioanalyser. The final libraries were pooled in equimolar amounts using Qubit and Bioanalyser data. Size distribution was assessed using the Fragment analyzer and equimolar pooling of the final libraries was performed. The final pools were cleaned-up to remove primer and adaptor dimers and assessed on the Bioanalyzer. The quantity and quality of the pools were assessed by the Bioanalyzer and subsequently by qPCR using the Illumina Library Quantification Kit from Kapa on a Roche Light Cycler LC480II according to the manufacturer’s instructions. Following calculation of the molarity using qPCR data, template DNA was diluted to 300pM and denatured for 8 minutes at room temperature using freshly diluted 0.2 N sodium hydroxide (NaOH) and the reaction was subsequently terminated by the addition of 400mM TrisCl ph = 8. To improve sequencing quality control 1% PhiX was spiked-in. The libraries were sequenced on the Illumina NovaSeq 6000 platform following the XP workflow on 3 lanes of an S4 flow cell, generating 2 × 150 bp paired-end reads. The raw fastq files generated by Illumina HiSeq and Novaseq were trimmed to remove Illumina adapter sequences using Cutadapt v1.2.1(29). The option “−O 3” was set, so the that 3’ end of any reads which matched the adapter sequence with greater than 3 bp was trimmed off. The reads were further trimmed to remove low quality bases, using Sickle v1.200 (30) with a minimum window quality score of 20. After trimming, reads shorter than 10 bp were removed.

### Differential expression analysis

Hisat2 v2.1.0 [40] was used to map the trimmed reads on the *Cavia porcellus* reference genome assembly with known splice sites (release-110) downloaded from the Ensembl FTP site. The alignments were used for calculating read counts per gene using featureCounts v2.0.0 [41] with a guide Ensembl GTF (release-110). The raw counts generated from featureCounts were imported into the R v3.4.1 environment to carry out differential expression analysis using edgeR v3.42.4 [45]. Differentially expressed genes with an FDR < 0.05 and an absolute log2 fold change > 1 were reported. The GO enrichment in biological process for the differentially expressed genes was performed using enrichGO function in clusterProfiler v4.8.3 [42] to query the Mustela putorius GO database (object: AH112154) via AnnotationHub v3.8.0. GO terms with q-value < 0.05 were considered significantly enriched and top 10 most significant categories of each cluster was plotted.

### de novo assembly of Lassa mammarenavirus strain GA391 genome.

The Lassa mammarenavirus strain GA391 genome was composed of segment S (GenBank accession no. OL774861.1) and segment L (GenBank accession no. OL774860.1). Since the GenBank segment L sequence is not full length, the partial GenBank segment L sequence was used as trusted contigs as in input into the SPAdes assembler (v3.15.5) [43] for *de novo* assembly with “rnaspades.py” script and default setting.

### Consensus virus genomes and nucleotide frequencies

The reads unmapped to the *Cavia porcellus* reference genome assembly were extracted by bam2fastq (v1.1.0) and then mapped on segment S (GenBank accession no. OL774861.1) and *de novo* assembled segment L of Lassa mammarenavirus strain GA391 genome using Bowtie2 v2.3.5.1 [40] by setting the options to parameters “--local -X 2000 --no-mixed”, followed by Sam file to Bam file conversion, sorting, and removal of the reads with a mapping quality score below 11 using SAMtools v1.9 [44]. After that, the PCR and optical duplicate reads in the bam files were discarded using the MarkDuplicates in the Picard toolkit v2.18.25 (http://broadinstitute.github.io/picard/) with the option of “REMOVE_DUPLICATES = true”. The resultant Bam file was processed by Quasirecomb v1.2 [45] to generate read coverage of each nucleotide site and a phred-weighted table of nucleotide frequencies. The phred-weighted table was parsed with a custom perl script to generate a dominant genome sequence as our previous description [46]. The dominant genome sequence was then used as a template in the second round of mapping to generate a consensus genome (site coverage > 10). With this method, we consensus genome sequence of source virus and passage samples.

### Minor variation of amino acid

The minor variations of amino acid in the genes of virus were called as our previous description [47]. Reads (unmapped on *Cavia porcellus* genome) were aligned to the consensus source virus genome sequence using Bowtie2 with the parameter of “--local -X 2000 --no-mixed”. The Bowtie2 outputs were processed in the same way as above to generate a Bam file without read duplication. This Bam file was then processed by diversiutils script in DiversiTools (http://josephhughes.github.io/btctools/) with the “-orfs” function to generate the number of amino acid change caused by the nucleotide deviation at each site in protein. In order to distinguish of low frequency mutations from Illumina sequence errors, the diversiutils used the calling algorithms based on the Illumina quality scores to calculate a P-value for each mutation at each nucleotide site [48]. The amino acid change was then filtered based on the P-value (< 0.05) and amino acid site coverage (> 5) to remove the low frequency mutations from Illumina sequence errors.

## Data Availability

All data are available within the manuscript with relevant accession numbers accessed from the National Center for Biotechnology Information (NCBI) within the text. The Illumina sequencing data are available under NCBI project accession number: PRJNA1237606.
